# Social Attitude to COVID-19 and Influenza Vaccinations after the Influenza Vaccination Season and between the Second and Third COVID-19 Wave in Poland, Lithuania, and Ukraine

**DOI:** 10.3390/ijerph19042042

**Published:** 2022-02-11

**Authors:** Tomasz Zaprutko, Yuliia Kremin, Michał Michalak, Jurga Bernatoniene, Lucjusz Zaprutko, Nataliia Hudz, Aleksandra Stolecka, Julia Cynar, Katarzyna Niewczas, Józefina Sprawka, Patrycja Skorupska, Joanna Wróbel, Piotr Ratajczak, Dorota Kopciuch, Anna Paczkowska, Krzysztof Kus, Bohdan Hromovyk

**Affiliations:** 1 Department of Pharmacoeconomics and Social Pharmacy, Poznan University of Medical Sciences, 7 Rokietnicka Street, 60806 Poznan, Poland; p_ratajczak@ump.edu.pl (P.R.); dorota.koligat@gmail.com (D.K.); aniapaczkowska@ump.edu.pl (A.P.); kkus@ump.edu.pl (K.K.); 2 Department of Organization and Economics of Pharmacy, Danylo Halytsky Lviv National Medical University, 75 Pekarska Street, 79010 Lviv, Ukraine; kremin_julia@i.ua (Y.K.); hromovyk@gmail.com (B.H.); 3 Department of Computer Sciences and Statistics, Poznan University of Medical Sciences, 7 Rokietnicka Street, 60806 Poznan, Poland; michal@ump.edu.pl; 4 Department of Drug Technology and Social Pharmacy, Medical Academy, Lithuanian University of Health Sciences, Eiveniu 4, LT-50161 Kaunas, Lithuania; jurga.bernatoniene@lsmuni.lt; 5 Department of Organic Chemistry, Faculty of Pharmacy, Poznan University of Medical Sciences Poznan, 6 Grunwaldzka Street, 60780 Poznan, Poland; zaprutko@ump.edu.pl; 6 Department of Drug Technology and Biopharmaceutics, Danylo Halytsky Lviv National Medical University, 75 Pekarska Street, 79010 Lviv, Ukraine; natali_gudz@ukr.net; 7 Student Scientific Society, Department of Pharmacoeconomics and Social Pharmacy, Poznan University of Medical Sciences, 7 Rokietnicka Street, 60806 Poznan, Poland; ola.stolecka@onet.pl (A.S.); julka.cynar@gmail.com (J.C.); kasia.niew@o2.pl (K.N.); jozia.sgz@gmail.com (J.S.); patrycja.skorupska99@gmail.com (P.S.); wrobel.joannaa@gmail.com (J.W.)

**Keywords:** pandemic, vaccination, influenza, COVID-19

## Abstract

The SARS-CoV-2 pandemic affected the entire world and contributed to severe health and economic consequences. A safe and effective vaccine is a tool allowing the pandemic to be controlled. Hence, we aimed to conduct a survey on vaccinations against seasonal influenza and COVID-19 in Poland, Lithuania, and Ukraine. We also evaluated societal attitudes towards influenza and COVID-19 vaccinations. Materials and methods: We conducted the study between December 2020 and May 2021. At the time, the countries subject to the research were between the second and third waves of the COVID-19 pandemic. We used an anonymous and self-designed questionnaire comprised of eleven closed-ended questions and a short socio-demographic section. The questionnaire was administered by direct contact or mainly (due to the COVID-19 pandemic) by e-mail or Facebook. Finally, we included 2753 answers from Poland, 1852 from Ukraine, and 213 from Lithuania. Results: Between 61% (Poland) and 72.9% (Ukraine) of the study participants have never been vaccinated against influenza (*p* < 0.05). Totals of 67.6% of the respondents in Poland, 73.71% in Lithuania, and 29.5% in Ukraine responded that they want to be vaccinated against COVID-19 (*p* < 0.05). Vaccine hesitancy was mainly related to worries about its side effects. There were also vaccine non-adopters in the study. In Ukraine, 67% of the respondents were clearly opposed to mandatory COVID-19 vaccines, compared to 41.7% in Poland and 30.99% in Lithuania (*p* < 0.05). Conclusions: There are still many people who present vaccine hesitancy or are opposed to vaccines. Thus, societal education about vaccination and the pandemic is crucial. Vaccine hesitancy or refusal might be related to vaccine origin. Shortages of influenza vaccines made it impossible to vaccinate those who were determined to be vaccinated. There is room for discussion of mandatory COVID-19 vaccinations.

## 1. Introduction

The SARS-CoV-2 pandemic has spread around the globe [1] with millions infected and hundreds of thousands dead [2]. Apart from health consequences, it presents a significant economic burden that cannot be underestimated [3]. Considering these facts, governments and decision-makers have to focus on the transmission of the virus. Hence, they had to regulate everyday life by limiting contacts and the mobility of the population [1], whilst simultaneously taking care of the economy.

With more knowledge about SARS-CoV-2, it became clear that the development of a vaccine is crucial to fight the pandemic and return to normal life [4]. Although vaccination is one of the most effective public health interventions [3,5], the challenge for policymakers is to encourage people to receive the vaccine and, in the case of COVID-19, to develop herd immunity [1]. Nonetheless, vaccine hesitancy and refusal are significant in many societies. Hence, the World Health Organization (WHO) recently placed them in the top ten threats to global health [5,6,7].

For example, even though a well-known vaccination against influenza leads to a lower incidence of seasonal influenza-related respiratory diseases, lower costs, and reduced deaths, mainly among the elderly population, the vaccination coverage differs between countries [8]. For instance, low coverage was recently observed in Poland: during the 2016–2017 influenza season, it was 3.3% for the entire population. Nonetheless, similar results were noticed in other Central European countries such as Romania (2.5%), Slovenia (3%), or Estonia (2.6%) [9]. In the UK, however, the vaccination coverage was about 50% [9], and this result was convergent with the coverage reached in the USA. In the 2019–2020 influenza season, it was 48.4% among Americans who were more than 18 years old [10]. However, during the next season (2020–2021) and in the time of the second and the third COVID-19 wave, influenza vaccination in elderly people increased in many countries, including, e.g., Italy (+10.7%), England (+8.5%), Poland (+3.3%), the Philippines (+3.0%), and the USA (+5.4%) [11].

According to the study conducted by Dror et al. [7], people vaccinated against seasonal influenza have a clear and strong acceptance of a COVID-19 vaccine. Without such acceptance, conspiracy theories against vaccines develop. People are more likely to believe them when they feel anxious, in a time of crisis, and when faced with global events with several consequences, such as the COVID-19 pandemic [12].

Hence, we decided to conduct a survey related to seasonal influenza and COVID-19 vaccinations in three Central and Eastern European countries (Poland, Ukraine, and Lithuania), of which Poland and Lithuania are European Union (EU) members and Ukraine is outside of the EU. Although these countries are neighboring and located in Central and Eastern Europe, they have essential differences. These are related to economic and geopolitical facets, and result from facts such as EU membership or population. Therefore, we assumed that the results of such analysis would be interesting both for the local and international readers or healthcare decision-makers. We aimed to evaluate societal attitudes towards influenza and COVID-19 vaccinations, and to examine the reasons for agreeing to or refusing to be vaccinated. We also intended to check the relationship between immunization against influenza in 2020–2021 and willingness to be vaccinated against COVID-19.

## 2. Materials and Methods

The study was conducted between December 2020 and May 2021, at a time when the countries which are the subjects of the research were between the second and third waves of the COVID-19 pandemic. We also tried to conduct the study in Latvia, but the obtained sample size was very low (*n* = 38), and we decided to exclude the country from further analysis.

An anonymous and self-designed questionnaire comprised of eleven closed-ended questions (six related to influenza and five to COVID-19) and a short socio-demographic section was one of the study tools. Questions concerned the same issues in each language version.

We authored the questionnaire based on observation of the pandemic development, the literature, and our own market and healthcare experiences. Due to this fact, ten potential participants evaluated the study tool and then translated it into local languages before the actual analysis. This made it possible to assess whether the study tool was straightforward. Ultimately, these questionnaires were not included, because after this preliminary part of the study, we amended and simplified some points of the questionnaire. For instance, in questions concerning obligatory vaccinations (influenza and COVID-19), we decided to offer an answer describing people “from the risk group” instead of presenting seniors, teachers, and medical staff individually. In question 7, we added information about the PCR test in square brackets in order to clarify the question and COVID-19 infection. The final questions content and possible variants of answers are presented in Table 1. In each country, we only collected answers from those ≥16 years old. Participation was voluntary and personal data were not requested. We offered no incentives for participation. The questionnaire was administered by direct contact or mainly (due to the COVID-19 pandemic) by e-mail or Facebook. Out of the 4849 gathered answers, we excluded 31 questionnaires (23 from Poland and 8 from Ukraine); for example, if no information was provided in the socio-demographic section or if participants did not answer some queries. In the case of online questionnaires, all responses were required to complete the study. Finally, we included 2753 answers from Poland, 1852 from Ukraine, and 213 from Lithuania.

## 3. Results

We decided to present most of the obtained results within tables, making this section, in our opinion, clearer and more reader-friendly. The leading groups of study participants were students or individuals about thirty years old (Table 2).

Between 61% (Poland) and 72.9% (Ukraine) of the study participants have never been vaccinated against influenza (question 1) and almost the same number of negative answers concerned the willingness to be vaccinated against influenza in autumn/winter of 2020/21 (question 2). Detailed results are presented in Table 3. Contrary to in Lithuania, in Poland and Ukraine we observed a statistically significant difference (*p* < 0.05) analysing for responses to question 1 if we considered whether the studies/work of our respondents are related to healthcare.

Compared to UE countries, in Ukraine 12% more participants (68.3%) claimed that vaccination against influenza should not be obligatory (question 6; *p* < 0.001). The “Agree, but only for those from the risk group” option was chosen by 29% of respondents in Poland, 27.70% in Lithuania, and 16.3% in Ukraine. In Poland and Ukraine, we observed strong statistical significance (*p* < 0.001) between studies/work in healthcare and acceptance of obligatory influenza and COVID-19 vaccinations. In Lithuania, this held true only for influenza.

Interestingly, in each country there was statistical significance (*p* < 0.001) between a patient’s history of vaccination against influenza and willingness to be vaccinated against COVID-19, as well as acceptance of an obligatory COVID-19 vaccination (in the latter case, *p* was 0.01 in Lithuania).

An analysis of COVID-19 infection history (confirmed by a PCR test) and willingness to be vaccinated against COVID-19 revealed statistical significance in Poland and Ukraine with *p* < 0.001 and *p* = 0.002, respectively. In Lithuania, *p* was 0.057. However, an analysis of COVID-19 infection history (confirmed by a PCR test) and the respondents’ opinions about the obligation to vaccinate against COVID-19 revealed statistical significance in Poland (*p* = 0.004) and Lithuania (*p* = 0.04), unlike Ukraine, where *p* was 0.223.

In the section of the questionnaire dedicated to COVID-19, most respondents indicated (question 7) that they have not tested positive for the coronavirus (84.2% in Poland; 69.9% in Ukraine; and 73.71% in Lithuania). Despite this, 67.6% of Polish respondents answered (question 8) that they would like to be vaccinated against COVID-19. In Lithuania, 73.71% of answers to that question were affirmative. However, in Ukraine, 29.5% of responses were positive, and the rest of the respondents would not like to be vaccinated against COVID-19. We observed a statistically significant difference in this question: Poland vs. Lithuania, and Ukraine with *p* < 0.001.

The difference was greater when it came to mandatory vaccination against COVID-19 (question 11) with 67% apparent opponents in Ukraine, compared to 41.7% in Poland, and 30.99% in Lithuania (*p* < 0.001). In both EU countries, more than 40% (41.7% in Poland, 44.6% in Lithuania) of responses were clearly positive. The “Agree, but only for those from the risk group” response was chosen by 17.2% of study participants in Poland, 24.41% in Lithuania, and 16.1% in Ukraine. We revealed a statistically significant difference for *p* < 0.05 between countries (excluding Poland vs. Lithuania) when comparing the answer “Agree” and “Agree, but only for those from the risk group” with *p* = 0.137.

Due to the severity of answers (multiple choice possibility) obtained in questions 3, 4, 5, 9, and 10, we decided to present the three most frequent responses to these questions (Table 4).

## 4. Discussion

The most important findings of our study are set forth below. There is low vaccination coverage against influenza in Poland, Lithuania, and Ukraine. However, a better coverage rate in the previous influenza season (2020/21) was made impossible by vaccination shortages. The willingness to be vaccinated against COVID-19 was distinctly lower in Ukraine than in the analysed EU countries. There are noticeably more opponents to obligatory vaccinations against COVID-19 and influenza in Ukraine (67%) than in Poland (41.7%) and Lithuania (44.6%). There is a correlation between influenza vaccinations and the acceptance rate for COVID-19 vaccinations.

According to the Patwardhan and Ohler study [13], the vaccine against influenza has already earned scepticism. It concerns the efficacy, disbeliefs and misconceptions about the safety and vaccine-hesitancy over the years. It may partially explain the low flu vaccine coverage in the countries analysed in the study [8,9]. Nevertheless, we revealed that in autumn 2020, when a vaccine against COVID-19 was still not available, almost 20% more respondents in Poland, 17% in Lithuania, and 12.5% in Ukraine wanted to be vaccinated against seasonal influenza than those who were actually vaccinated. For example, the interest in influenza vaccinations could have resulted from the fact that the flu vaccine was considered to afford protection against COVID-19 [14,15,16,17]. Considering that EU member states are trying to attain good influenza vaccination coverage, with an expected rate of 75% amongst the elderly population, for example [8], the influenza season was a great chance to boost the vaccination coverage in the analysed countries, bringing them closer to the level reached in the UK, or the USA [9,10], where a 75% coverage rate was exceeded during the 2020–2021 season [11]. However, the insufficient market stock of vaccines might be the result of drug shortages. This phenomenon arises from several facets such as disparities in drug prices among EU countries or the reverse traffic of medicines [18].

Nonetheless, it seems that all pharmaceutical market stakeholders had time to provide an adequate stock of flu vaccines in autumn 2020. In the societies of the analysed countries, an interest in vaccinations was already observed during the first wave of the pandemic and in the months preceding autumn. Notably, better coverage in immunization against seasonal influenza would also positively impact the attitude to COVID-19 vaccinations. Studies have shown that the best predictor of the uptake of a pandemic vaccine is the administration of an influenza vaccine in the previous season [1,7,14]. This was also confirmed by our study. Moreover, Patwardhan and Ohler [13] revealed that patients who received seasonal influenza vaccinations were less likely to develop symptomatic and severe infections. In turn, Conlon et al. [14] noticed that the percentage of patients who (4.0%) tested positive for COVID-19 was 0.9% lower among those who had received an influenza vaccine between 1 August 2019 and 15 July 2020. Conlon et al. and Zanettini et al. also observed an association between flu vaccination and reduced COVID-19 mortality, decreased need for intensive care treatment, as well as invasive respiratory support [14,16].

Nonetheless, vaccine availability does not guarantee a sufficient population vaccination rate, as shown by vaccine hesitancy [7]. Disbeliefs, conspiracy theories, and vaccine hesitancy became a real challenge in the fight against COVID-19. We also revealed that in the analysed countries, the main reason for unwillingness to be vaccinated against COVID-19 was fear of vaccine safety. This was followed by general disbelief in vaccines or a conviction that one is not in a risk group. The above confirms the need for uninterrupted societal education on COVID-19 and vaccinations [7]. It is also concurrent with the results of our study, where participants (Poland) claimed that better knowledge about the vaccine composition and production process, as well as more extended market availability (Ukraine, Lithuania), may change their negative attitude towards COVID-19 vaccinations.

Contrary to respondents from Ukraine (29.5%), almost 68% in Poland and 74% of participants in Lithuania claimed that they would receive a COVID-19 vaccine if it only were to become available [19]. The difference observed between EU countries and Ukraine may result from the fact that in Poland, 61.2% of study participants were engaged in the provision of healthcare. In Lithuania, it was 53.5% and in Ukraine 38.4%. Moreover, the reluctance to be vaccinated against COVID-19 in Ukraine may also result from the geopolitical facets and the vaccine origin. Many Ukrainians present pro-Western attitudes, which are also visible in their approach to vaccines. Almost 70% of them do not trust the Chinese vaccine, and more than 80% do not trust the Russian one, whereas their acceptance for vaccines from the US or UK is higher [20,21]. Nonetheless, the acceptance rate gained in EU countries participating in our study is convergent with the result obtained in the USA by Mercadante and Law [19]. However, in the Middle Eastern population, COVID-19 vaccination acceptance was lower than in the analysed EU countries, but still higher than in Ukraine [3].

These differences may also result from the impact of social media on attitudes towards vaccines. Disbeliefs, misinformation, and unsubstantiated rumours, both about the pandemic and vaccination against COVID-19, have already begun to emerge on social media before the actual release of an effective vaccine [5]. Moreover, Earhshaw revealed that people who believed conspiracies trusted misinformation about COVID-19 from social media to a greater extent than participants who disbelieved them [12]. Besides, social media recently became a primary and trustworthy source of health information [22,23], making it an unstoppable communication tool.

Extensive anti-vaccine content is often shared across social media. Exposure to such misinformation may directly fuel vaccine hesitancy [5]. Betsch et al. revealed that contact with vaccine-critical websites and blogs negatively impacts the intention to vaccinate [24]. Besides, amongst the top YouTube videos related to COVID-19 and the coronavirus, 27.5% contained non-factual information and had tens of millions of views [5]. Considering that the study participants were young adults in general, they were more likely to use social media. This may explain the worries presented by vaccine non-adopters who participated in our study. Respondents were mainly afraid of vaccine safety. They also believed that they were not in a risk group or did not believe in vaccines. This confirms the need for several actions [8] to overcome barriers of mistrust in the community and boost the role of trustworthy information presented by experts and health professionals about vaccine composition, manufacture, administration, and safety [25].

Although mandatory vaccination against COVID-19 might be considered controversial, we revealed a surprisingly high acceptance rate for it in Poland and Lithuania, with 41.7% and 44.6% in favour, respectively. In Ukraine, the acceptance rate was lower. A few months after we finished the study (April 2021), some countries introduced mandatory vaccinations. Interestingly, in Tajikistan and Turkmenistan, vaccination against COVID-19 is obligatory for all adults. In countries like France, Italy, or the US, vaccines are compulsory for certain adults and sometimes in certain areas (USA, Russia) [26]. In many countries, including Poland, there are consultations and political discussions concerning mandatory vaccination for frontline health and social care staff [27]. Obligatory vaccinations for selected professional groups seem to have a higher acceptance rate in communities. In our study, the mandatory vaccination only for those from a selected professional risk group (e.g., physicians, pharmacists) was chosen by 17.2% more Polish and 24.41% more Lithuanian participants than those who declared their support to obligatory vaccinations in general. In Ukraine, however, this option was accepted by 16% of respondents. Although mandatory COVID-19 vaccinations imply serious ethical hurdles, widespread acceptance of COVID-19 vaccines is crucial for achieving herd immunity and ending the global pandemic [28]. Thus, vaccination campaigns should focus on societal education in order to overcome the disbeliefs and vaccination mistrust created by very active groups which frequently use social media, for example, to promote non-factual information.

### Limitations

It would be valuable to include more seniors in our study. However, it was difficult to add them into the study group due to limited contact possibilities; for example, older people are frequently unfamiliar with online communication. Although the discrepancy between the number of answers included in the following countries may seem to influence the results, there are differences in the population of analysed countries: 38 million, 42 million, and 2.8 million inhabitants in Poland, Ukraine, and Lithuania respectively. Thus, the presented difference seems to justify the comparability of the gathered questionnaires. Besides, we are aware that the study could benefit from the participation of respondents from other EU (Western) and non-EU countries. It would also be interesting to ask about personal beliefs on given vaccinations using their commercial names, such as “Sputnik”, “AstraZeneca”, or “Moderna”.

## 5. Conclusions

Although the COVID-19 pandemic has significant health and economic consequences, many people present vaccine hesitancy or are opposed to vaccines. Thus, societal education is needed on the severity of possible side effects, their comparison with the side effects of other medicines (the severity of blood clots after contraceptives), and the vaccine production process. It will also help in suppressing the scope of non-factual information, such as that coming from social media, for example. Vaccine hesitancy or refusal might also be related to vaccine origin and political facets. Shortages of influenza vaccination made it impossible to vaccinate those who were determined to be vaccinated. Considering that vaccination against seasonal influenza might also afford protection against COVID-19, full and fair access to vaccinations should be a priority for healthcare decision-makers. There is room for discussion about mandatory COVID-19 vaccinations, even for selected groups of workers.

## Figures and Tables

**Table 1:** Questions content and possible answers.

Question	Question Content	Possible Answers
1 ^#^	Have you ever been vaccinated against influenza?	Yes, annually/Yes, occasionally/No
2 ^#^	Willingness to be vaccinated against influenza in the season 2020/21	Yes, and I was vaccinated/Yes, but the vaccine was not available/No
3 ^#^	If your answer was “affirmative” in the previous question, please indicate why? If the answer was “no” please skip this question and go to question 4.	Multiple choice possibility
4 ^#^	If you answered “no” in question 2 please indicate why?	Multiple choice possibility
5 ^#^	If you answered “no” in question 2 please indicate what could change your decision?	Multiple choice possibility
6 ^#^	Do you think that vaccination against influenza should be obligatory?	Agree/Agree but only for those from the risk group/Disagree
7 ^^^	Have you been infected with the coronavirus (infection confirmed by the PCR test)	Yes, and the course of illness was severe/Yes, and the course of illness was mild/Yes, and the course of illness was asymptomatic/No
8 ^^^	Would you be vaccinated against COVID-19?	- Yes/no
9 ^^^	If you answered “disagree” in the previous question, please indicate why? If the answer was affirmative, please skip this question and go to question 5.	Multiple choice possibility
10 ^^^	If you answered “disagree” in the previous question, please indicate what could change your decision?	Multiple choice possibility
11 ^^^	Do you think that vaccination against COVID-19 should be obligatory?	Agree/Agree but only for those from the risk group/Disagree

^#^ section related to influenza; ^^^ section related to COVID-19.

**Table 2:** Structure of the study group.

Country	Age Range *			Sex		Place of Residence			Level of Education			Work/Education Related to Healthcare ^^^
	1 ^#^	2 ^#^	3 ^#^	Female	Male	1 ^#^	2 ^#^	3 ^#^	1 ^#^	2 ^#^	3 ^#^	Yes
Poland	18–26 (65.5%)	36–45 (9.9%)	27–35 (9.8%)	73.8%	26.2%	City > 500 thousands of residents (35%)	City with 100–500 thiousands of residents (20.9%)	City < 50 thousands of residents (19.6%)	Student (47.5%)	Higher (38.2%)	Secondary (10.31%)	61.2%
Ukraine	27–35 (32.5%)	36–45 (29.7%)	46–60 (16.8%)	82.3%	17.7%	City > 500 thousands of residents (49.5%)	City with 100–500 thiousands of residents (19.7%)	City < 50 thousands of residents (14.4%)	Higher (83%)	Student (7%)	Secondary (6.1%)	38.4%
Lithuania	18–26 (39.44%)	36–45 (17.37%)	27–35 (14.55%)	72.77%	27.23%	City with 100–500 thiousands of residents (41.31%)	City < 50 thousands of residents (19.72%)	City with 50–100 thiousands of residents (14.08%)	Higher (60.56%)	Student (23.0%)	Secondary (7.98%)	53.5%

^#^ 3 most frequent answers; * There were 5 possible age ranges. We presented 3 with the highest percentage of participants in each country; ^^^ Statistically significant difference: Poland vs. Lithuania, and Ukraine with *p* < 0.001.

**Table 3:** Willingness to be vaccinated against influenza.

Country	Have You Ever Been Vaccinated against Influenza? ^#^			Willingness to Be Vaccinated against Influenza in the Season 2020/21 ^#^		
	Yes, Annually	Yes, Occasionally	No	Yes, and I Was Vaccinated	Yes, but the Vaccine Was Not Available	No
Poland	10.7%	28.3%	61%	16.4%	19.6%	64%
Ukraine	10.7%	16.4%	72.9%	14.8%	12.5%	72.7%
Lithuania	10.33%	20.66%	69.01%	17.84%	16.9%	65.26%

^#^ Statistically significant difference: Poland vs. Lithuania, and Ukraine with *p* < 0.001.

**Table 4:** Answers related to multiple choice questions (3, 4, 5, 9, and 10).

Country	Question 3			Question 4			Question 5			Question 9			Question 10		
	1 ^#^	2 ^#^	3 ^#^	1 ^#^	2 ^#^	3 ^#^	1 ^#^	2 ^#^	3 ^#^	1 ^#^	2 ^#^	3 ^#^	1 ^#^	2 ^#^	3 ^#^
Poland	“I feel safe being vaccinated against influenza” (80.69%)	“employers cover the costs of vaccination” (13.3%)	“I know that it is important” (11.4%)	“I am not in a risk group” (48.9%)	“I am afraid of side effects” (25.3%)	“I have never got influenza (15.1%)	“Being in a risk group” (46.25%)	“Worsening of the pandemic course” (37.8%)	“Better access to vaccines” (24.2%)	“I am afraid of COVID-19 vaccines safeness” (76.4%)	“I am not in a risk group” (28.6%)	“I am afraid of vaccines costs” (23.4%)	“If it will be necessary” (59.1%	“Getting knowledge about the process of production and vaccine composition” (25.8%)	“Longer market availability” (13.4%)
Ukraine	“I feel safe being vaccinated against influenza” (59.3%)	“I am in a risk group” (19.1%)	“in the past, my relatives or I was seriously ill due to influenza” (10.9%)	“I am anti-vaccines” (41%)	“I am afraid of side effects” (32%)	“I am not in a risk group” (12.5%)	“Nothing can change my decision” (61.9%)	“Being in a risk group” (9.7%)	“Worsening of the pandemic course” (7.8%)	“I am afraid of COVID-19 vaccines safeness” (34.3%)	“I do not believe in vaccines” (32.4%)	“I am not in a risk group” (13.9%)	“Nothing can change my decision” (63.2%)	“Longer market availability” (18.1%)	“Worsening of the pandemic course” (4.7%)
Lithuania	“I feel safe being vaccinated against influenza” (26.29%)	“employers cover the costs of vaccination” (5.63%)	“I am in a risk group” (3.75%)	“I am not in a risk group” (25.35%)	“I am not interested in vaccination” (25.35%)	Other (answer not specified) (10.33%)	“Being in a risk group” (26.29%)	“Nothing can change my decision” (21.13%)	“Serious influenza complications in my family” (19.72%)	“I am afraid of COVID-19 vaccines safeness” (50%)	“I do not believe in vaccines” (25.0%)	“I am not in a risk group” (25.0%)	“Nothing can change my decision” (55.36%)	“Longer market availability” (48.21%)	“Severe course and complications after COVID-19 in my family” (23.21%)

^#^ 3 most frequent answers.
